# Surgery

**Published:** 1859-07

**Authors:** Samuel W. Gross


### Bi-Monthly Abstracts;

OR, BI-MONTHLY NOVELTIES IN MEDICAL, PATHOLOGICAL, SURGICAL, OBSTETRICAL, PHYSIOLOGICAL, AND CHEMICAL SCIENCE.

### SURGERY.

BY SAMUEL W. GROSS, M.D.

I.—*Congenital Extrophy of the Urinary Bladder, and its Complications, successfully treated by a new Plastic Operation.* By DANIEL AYRES, M.D. (Pamphlet reprint from the New York Medical Gazette, February, 1859.)

IN this paper Dr. Ayres relates a case of extrophy of the bladder, complicated with prolapse of the uterus, occurring in a female twenty-eight years of age, who was admitted into the Long Island College Hospital, on the first of November, 1858. Four months previously she had been delivered, at the full term, of a well-developed child, which died during parturition, this act having been performed with no great difficulty. Soon after her recovery the uterine complaint made its appearance, for which she had in vain been wearing different forms of pessaries. She was of a weak, sickly appearance, and her general health was not good.

The separation of the pubic symphysis was estimated at about three inches, and the bladder formed an oval, bright red, sensitive tumor, two inches in length by an inch and a quarter in breadth. The integuments between the protruded viscus and the ensiform cartilage were devoid of hair, and were red, soft, and sensitive. The labia majora were large, and lost themselves in either thigh, the nymphæ projected on each side of the vulva, and there was an absence of the clitoris and urethra.

To relieve this deformity, Dr. Ayres proposed—"First, to form an anterior wall for the exposed bladder; second, to restore the urinary canal; third, to establish the anterior fourchette of the vulva; fourth, to supply means to prevent the prolapse and to correct the renal secretions."

To fulfill these indications, on the sixteenth of November Dr. Ayres performed a plastic operation, which, as the result shows, was not only very ingenious, but successful. It consisted in raising a large flap from the anterior and superior portion of the abdomen, and attaching it to the surrounding parts by means of the interrupted suture. To retain the parts perfectly in place, adhesive strips, compresses, and a bandage were applied. The flap covered the bladder, with the exception of a triangular space at its lower portion, which was left exposed for another operation. On the fourth day all the sutures were removed, and the parts were found to have united, with the exception of a small spot the size of a ten-cent piece. On the seventh of December this had nearly healed by granulation, when the second operation was performed in such a manner as to establish the anterior fourchette of the vulva and to form a urinary canal.

One month after the operation the patient visited the hospital, having walked two miles. The parts were firmly united, the bladder was completely covered, the vulva was diminished in size, and the prolapse of the uterus had disappeared, giving place to a prolapse of the anterior fold of the vagina, which was, however, easily supported by a light pessary.

[While we congratulate Dr. Ayres upon the happy result of this case, we must state that the operation is not novel, plastic operations having been performed for the relief of this deformity by several surgeons, including Pancoast, Langenbeck, and Dieffenbach. Dr. Ayres does not seem to be aware of the case of Professor Pancoast, who operated on the patient at the clinic of the Jefferson Medical College, in February, 1858. As this case has never been published, we append a few notes of its history.

The patient was a male, from Michigan, twenty-eight years of age, and was short and apparently stunted in growth. The external genital organs were more than usually developed, and there was a very large inguinal hernia on the right side extending down into the scrotum. The walls of the bladder, when fully protruded, formed a tumor four inches in breadth and extending vertically from about an inch below the umbilicus to the urethral groove on the top of the penis. When the lower part of this tumor was raised, the orifices of the ureters and a rudimental caput gallinaginis could be perceived. In order to prevent this protrusion of the bladder, which caused the patient so much pain and suffering as to disable him from following any occupation, the following operation was performed:—The bladder was pushed back into the cavity of the abdomen and held by pressure on a sponge. A large flap was raised on either side, with its base to the bladder and notched at its outer side, its lower end extending on the side of the penis to near the glans. These were of such a shape as to meet exactly in the middle line and form a sort of dome over the cavity of the bladder, the cutaneous surface turned toward the bladder, and the raw tissue left to cicatrize and form a new skin. This raw surface was kept covered with a mixture of collodion and glycerine, so that the air should be excluded and the cicatrization be made to go on so slowly as not to interfere with the circulation in the flaps. The flaps were united in the middle line by the tongue and groove suture, and transversely where the notches in the flaps came together, by the Bozeman suture. Union took place throughout by first intention, but subsequently opened under the pressure of the abdominal muscles, near the pubes. The extensive wound made by raising the flaps, which on the right side included the outer covering of the hernial tumor, was closed as much as possible by drawing the edges of the wound together and the sliding round of some secondary flaps, raised from the adjoining surface. A large amount of raw surface was left, however, for cicatrization, and kept covered from the air. Nearly the whole of the skin which was taken to form a dome for the bladder was covered with an unusual strong growth of hair; this was closely shaved off. From what Dr. Pancoast had observed in other cases, he had no hesitation in turning the roots of these hairs toward the mucous cavity of the bladder, as he was sure their contact with the urine would not only prevent the reproduction of the hair, but cause the bulbs to be shed and discharged, an opinion which the result verified.

When the union and cicatrization of parts were complete, the hernia was found entirely cured by the cicatricial contraction. The bladder was completely held back, and instead of the large gap above the pubes, through which the bladder formerly bulged in a large mass, there was an opening, just above the penis, an inch long and a quarter of an inch wide, through which, when an effort with the abdominal muscles was made, the bladder could be made to project slightly. The patient was dismissed from the hospital, with his condition in every way vastly improved, and with the expectation of returning the ensuing fall to have the remaining small opening closed, and, if possible, have the epispadial groove on the back of the penis converted into a urethral tube. This, however, was prevented by the death of the patient, two months and a half subsequently, from epidemic typhoid pneumonia.]

II.—1. *A Case of Popliteal Aneurism successfully treated by Flexion of the Knee-joint.* By ERNEST HART, Esq.

2. *Report of a Case of Popliteal Aneurism successfully treated by Continued Flexion of the Knee-joint.* By ALEXANDER SHAW, Esq. (Papers read before the Royal Medical and Chirurgical Society of London. Medical Times and Gazette, May 7, 1859, p. 482.)

These interesting surgical contributions contain the results of two cases of popliteal aneurism treated by flexion of the knee-joint, a procedure which recommends itself on account of its simplicity and success. The use of the knife is evidently giving way to the well-directed efforts of surgeons of the present day in curing this formidable disease by various forms of compression, and in doing away with the danger which, until this method came into general adoption, always environed this class of cases.

1. Mr. Hart's case occurred in a man, forty-one years of age, who had well-marked symptoms of an aneurism, which was the size of a small apple, in the external and inferior portion of the right popliteal space. In making an examination of the parts the author discovered that when the leg was fully flexed the pulsation in the tumor ceased entirely, and the thrill nearly so. He therefore determined to maintain the limb in this position, with the hope of effecting a cure. After a week's preparatory treatment the leg was bandaged from the toes to the knee, the tumor not being included, and being completely flexed upon the thigh was retained in this position by the application of a stout roller. At the end of forty hours considerable solidification was found to have taken place in the tumor; and on the fifth day, when it was solid and without pulsation or thrill, the limb was eased and confined at a right angle. On the seventh day the leg was slung and the patient was allowed to move about, and on the twelfth day the limb was extended and the patient walked about, although the knee was somewhat stiff from the confinement in its constrained position. The tumor gradually diminished in size, and at the end of three months it had nearly entirely disappeared.

During the treatment but little inconvenience and no positive pain were experienced, the patient being thin and young, circumstances favoring this method.

Mr. Hart ascribes the cure to the obstruction of the circulation from the acute flexion of the artery, and the direct pressure exerted upon the sac causing the formation of active clots.

2. The patient of Mr. Shaw was a man, thirty years of age, who had a recent aneurism, the size of a lemon, in the left popliteal region. The knee was placed in the flexed position by appropriate apparatus, with the result of stopping the pulsation. The dressings were removed on the fourth day, when the tumor was found to have diminished one-third, its walls were more firm, and its pulsations less violent. Between the third and fourth week the pulsations were very feeble, the tumor felt very solid, and its volume was much reduced. On the thirty-eighth day the pulsations had ceased and the tumor was about the size of a walnut. The knee in the mean time had become stiff, but gradually recovered its functions. For the first ten days some pain was experienced, and a slight swelling of the knee required the application of a lead lotion. On the fifty-sixth day the patient was discharged cured, with complete use of his knee.

In the discussion which ensued upon these cases, Mr. Fergusson stated that two cases had occurred in King's College Hospital, one of which failed, the patient becoming weary of the treatment, while the other was successful.

Mr. Birkett reported three cases in which the treatment had been unavailing, probably on account of its having been employed for an insufficient length of time.

Mr. Shaw mentioned another case of an old man with a large popliteal aneurism, in which he had employed the same procedure, but without success.

From the above facts it will be perceived that eight cases have been treated by simple flexion of the knee-joint, three being complete cures; a sufficient number to recommend it to the profession and justify its further trial.

III.—1. *Treatment of Hydrocele by Electricity.* By DR. RODOLFI. (Gaz. Medic. Lombard, and Medizinische Neuigkeiten, September 25, 1858.)

2. *Particular Method of Curing Hydrocele almost instantaneously, and without an Operation.* By M. Pétrequin. (Archives Générales, March, 1859.)

1. Sustained by the fact of the galvanic current decomposing fluids, Dr. Rodolfi has applied this means to the treatment of serous effusions into the sac of the tunica vaginalis, which becomes so changed under the influence of electricity as to arrest the abnormal secretion.

In support of this novel treatment the author reports four cases of hydrocele, three being radical cures. The subjoined case will serve as an illustration of the treatment.

A cabinet-maker, thirty years of age, had suffered for fifteen months from a hydrocele of the left side, which had attained the size of the head of a newly-born child. Being placed in a convenient position upon an elevated bolster, with the thighs slightly flexed and widely separated, two platinum needles were introduced, as far as possible from each other, into the scrotum, and connected with a galvanic battery. In ten minutes the poles were reversed, and after sixteen minutes, were withdrawn from the scrotum. To the complete astonishment of all present the hydrocele had disappeared, and in two days the patient left the hospital completely cured. The author saw the case several months afterwards and convinced himself that the cure was a radical one.

The apparatus employed by Dr. Rodolfi consists of the platinum needles, of the screws, conducting wires, and battery of Bunsen, or Daniel, composed of from two to four simple circles; the latter apparatus being preferable on account of the acid not becoming troublesome. The operation consists of four stages—First, introduction of the needles; second, connection of the battery; third, change of the poles; and fourth, withdrawal of the needles. The needles should be guarded by small rings a short distance from their points, in order to prevent them from penetrating too deeply. During the whole proceeding it is necessary to compress the scrotum slightly, and be careful lest the vermicular movements of the dartos force the needles out of place.

2. M. Pétrequin, of Lyons, in a note to the *Académie des Sciences*, January 17, 1859, states that, in view of the great changes produced by electricity upon absorption and secretion, he conceived the idea of applying this fluid to the treatment of dropsies, and particularly of hydroceles. In 1849 he operated upon a patient, forty-five years of age, who had an old, voluminous hydrocele. The poles of a Bunsen's battery were applied, one to the base, the other to the summit of the tumor, for about half an hour. The next day the hydrocele had disappeared, and no relapse had taken place at the end of a month.

IV.—*On the Treatment of Chronic Hydrocephalus by Injections of Iodine.* By DANIEL BRAINARD, M.D. (Chicago Medical Journal, April, 1859.)

Dr. Brainard, in view of the general futility of the treatment of this affection by tapping, compression, and other methods, calls the attention of the profession to the injection of iodine, in the form of Lugol's solution, into the lateral ventricles of the brain for the relief of a preternatural secretion of serum in these cavities. His reasons for this recommendation, which, at first sight, might seem a rash one, may be stated to be that chronic hydrocephalus is an encysted dropsy dependent upon an increase of serum in the ventricles of the brain, which has no connection whatever with the cerebro-spinal fluid, the sac for the fluid being formed by the ependyma, or lining serous membrane of the ventricles. Since, therefore, iodine proves so successful in other serous cysts, and the sac of chronic hydrocephalus is of limited extent, its injection with iodine would have the effect, as in hydroceles, of altering the secreted fluid, and changing the secernent membrane. The lining membrane of the ventricles is devoid of nerves, red vessels, and irritability, and the presence, therefore, of the injection produces neither pain nor spasm. After hydrocele the author considers cases of chronic hydrocephalus most suitable for the use of this remedy, and to show that it is not only not dangerous, but justifiable, he adduces two cases, of which the following are brief abstracts:—

CASE I.—Dr. Tournesko, of Bucharest, drew off eleven ounces of fluid from the head of a child, two months old, the head measuring twenty inches. At the expiration of twenty-four hours the head had refilled, when a large-sized trocar was introduced through the side of the coronal suture into the lateral ventricles to the depth of two inches, and twenty-four ounces of fluid drawn off. Sixteen grains of iodine in three drachms of alcohol were then injected, the immediate effects upon the child being but slight. Twenty days afterwards the child was in good health, and the head was of a natural size. At the end of fifteen days more, the patient was in the same satisfactory condition.

CASE II.—Was a child, who, when first seen by the author, was four days old, and had chronic hydrocephalus along with bifid spine. The head measured nineteen inches in circumference, the upper portions of the parietal and occipital bones were wanting, and the head was hot, tense, covered with large veins, and rapidly increasing in size. The spinal tumor in the lumbar region was three inches in length, and one and a half inch in breadth. When the child was four weeks old, Dr. Brainard injected the latter with one-sixteenth of a grain of iodine and one-eighth of a grain of iodide of potassium in half a drachm of water, with the result of obtaining a cure at the expiration of six months. As the tumor of the back diminished, the size of the head increased, and it was, therefore, treated without delay. During a period of seven months the head was injected nineteen times with iodine, in the form of Lugol's solution, the quantity ranging from one-sixteenth to twelve grains. At each time of the injection of the larger quantity, twitching of the limbs and vomiting were produced, as well as once when nine grains were used, and once when six grains were thrown in, the latter of which was followed by convulsions, in which the child died. This case, therefore, showed that iodine is innocent when employed in this way, and although it did not result in a cure, it probably had the effect of prolonging life.

In view of the difficulty of carrying a sufficient quantity of iodine into the ventricles to excite a proper amount of action without leaving so much as will produce, by absorption, bad effects upon the system, Dr. Brainard recommends, First, to use a trocar of a certain size, as that employed for hydrocele; second, compress the head, and draw off as much fluid as practicable; third, inject a solution of iodine of such strength as that, when mixed with the fluid remaining in the ventricles, it will be of the strength of half a grain of iodine to the ounce of liquid; fourth, throw this through a gum-elastic tube, carried through the canula into the ventricle; fifth, inject the fluid first drawn off through the same tube to expel the solution.

V.—*On Extirpation of Fibroid Tumors of the Uterus.* By PROFESSOR B. LANGENBECK. (Deutsche Klinik, I., 1859.)

Professor Langenbeck, in this paper, after making some general remarks upon the seat, texture, and want of success of the medical treatment of fibroid tumors of the uterus, discusses the propriety of their removal, a procedure which has been practiced by Lisfranc, Amussat, Boyer, Kiwisch, and Simpson, with variable success. He is of the opinion that the operation is contra-indicated in those cases in which the tumor is seated in those portions of the uterus covered by peritoneum, and that it is inadmissible in interstitial fibroid tumors of the fundus of the organ, on account of the impossibility of ascertaining, even by the most rigid examination, the distance of the tumor from the peritoneum, and the consequent liability of the membrane to inflammation, aroused by tearing out or pulling off the morbid growth. On the other hand, the operation is indicated, when, in young patients, it seems impossible to check the dangerous and exhausting hemorrhages, when the tumor occupies the anterior or posterior wall of the cervix, or one of the lips of the os, and is accessible to the knife. If the tumor is seated in the cavity of the uterus, and does not protrude at the os, the latter must, as an initiatory step, be sufficiently dilated by a sponge-tent. The patient is placed in the same position as that for lithotomy, and the external genitals are separated by a speculum. To facilitate the extirpation, it will be found useful to make light pressure upon the hypogastrium, and to push the uterus downward by two fingers introduced into the rectum. The operation is commenced by dividing the lip of the os and that portion of the neck which is opposite to the tumor, when the surgeon introduces his left index finger, if possible, so high as to enable him to reach the upper border of the growth, and then carries a straight knife along the finger, and divides with one stroke the mucous membrane and proper tissue of the uterus down to the substance of the tumor. The tissues are then carefully detached by means of the finger or the handle of the knife; firm, fibrous bands are cut through with a pair of strong scissors, and the whole tumor is thus gradually removed. When the adhesions are very strong, it will become necessary to render the tumor tense, by drawing it down with a vulsellum, while the finger, knife, or scissors separates it cautiously from its uterine connections. Professor Langenbeck reports three cases in which he has performed the operation.

VI.—*On the Treatment of Prolapsus Ani in Children.* By M. GUERSANT. (Behrend und Hildebrand's Journal für Kinderkrankheiten, 1859, 1, 2.)

In a small hospital in London, to which patients suffering from diseases of the rectum alone are admitted, prolapse of the anus is not treated by operation. According to Dr. Salmon, this disease can be cured, not only in children, but also in adults, by the evacuation of the bowels in the recumbent posture, as in this position the patient cannot strain much, thus, generally, preventing the descent of the intestine. After the observance of this treatment for some time, the relaxation of the parts, which permitted the prolapse, disappears, and a cure is effected. At the same time vegetable astringent injections are employed, although alum will be found useful. As a local application strychnia may also be mentioned. This was first employed successfully by Duchassoy, who made two or three very small blisters around the margin of the anus, and dressed the raw surfaces with the ointment of strychnia. Dr. Johnson treated, in 1854, two cases by the local application of the same remedy; he states that in the lighter and more recent forms of prolapse he obtained a cure by regulating the bowels, employing astringent injections, and administering tonics. In neglected cases, when the sphincter is much relaxed, strychnia may be of some use, but it is not, however, to be recommended, as it is unreliable in grave, and superfluous in light cases; it is, moreover, troublesome, and not without danger. Dr. Johnson considers cauterization after Guersant's method more valuable.

In the Metropolitan Free Hospital, Mr. Hutchinson employs the tincture of nux vomica, in very small doses, since he believes it imparts tone to the sphincters and walls of the intestines.

Sir Benjamin Brodie prescribes, internally, calomel and rhubarb, and uses injections of the dilute tincture of the chloride of iron.

Mr. Ashton maintains that in children suffering from this affection, the liver is principally to be attended to; and Mr. Curling recommends the use of cod-liver oil, after sufficient purgation.

Mr. Salmon treats recent cases in children in the following way: First, the patient must evacuate his bowels in no other but the recumbent posture; second, the prolapsed part is thoroughly moistened with a decoction of oak-bark and alum; third, internally, he employs calomel and rhubarb; when he orders, fourthly, tonics, and a nourishing diet.

When the usual remedies have been employed without effect, and the symptoms remain obstinate, M. Guersant advises cauterization of the skin and sphincter muscles with the hot iron. He does not cauterize the whole of the protruded mucous membrane, as was formerly done, as he considers the measure barbarous and hazardous. He proceeds, generally, in the following manner: The child is subjected to a certain diet, and shortly before the operation an injection is administered to clean out the lower bowel. The patient is placed upon his side, his thighs are flexed, and the protruded rectum reduced; an assistant drawing away one buttock, while the operator controls the other. The cautery is about the shape of that used by dentists, either curved or straight, and terminates in a small ball, which, above, runs out into a little point. It is applied at opposite points around the anus, and must penetrate the sphincter in order to be efficient. It is also necessary to draw the margins of the anus well apart on applying the cautery, and allow the small ball, after its point has penetrated the sphincter, to act upon the margins of the skin and mucous membrane. The patient must be placed under the influence of chloroform, and the iron be used at a white heat. If the rectum protrude during the operation, it should be pushed out of the way of the instrument. Cold-water dressings are the only applications made after the operation.

M. Guersant recommends this plan of treatment as an excellent, but not infallible means. In a few rare cases the children are cured in one day; more frequently, however, the prolapse returns in a few days, and the cure is not completed before the eighth or tenth day, when cicatrization has taken place. Sometimes a second cauterization will become necessary.

